# The influence of vitality forms on action perception and motor response

**DOI:** 10.1038/s41598-021-01924-w

**Published:** 2021-11-19

**Authors:** G. Lombardi, J. Zenzeri, G. Belgiovine, F. Vannucci, F. Rea, A. Sciutti, G. Di Cesare

**Affiliations:** 1 Department of Informatics, Bioengineering, Robotics and Systems Engineering (DIBRIS), University of Genoa, Genoa, Italy; 2 Cognitive Architecture for Collaborative Technologies Unit, Istituto Italiano di Tecnologia, Genoa, Italy; 3 Robotics Brain and Cognitive Sciences Unit, Italian Institute of Technology, Genoa, Italy

**Keywords:** Social behaviour, Neuroscience, Cognitive neuroscience

## Abstract

During the interaction with others, action, speech, and touches can communicate positive, neutral, or negative attitudes. Offering an apple can be gentle or rude, a caress can be kind or rushed. These subtle aspects of social communication have been named *vitality forms* by Daniel Stern. Although they characterize all human interactions, to date it is not clear whether vitality forms expressed by an agent may affect the action perception and the motor response of the receiver. To this purpose, we carried out a psychophysics study aiming to investigate how perceiving different vitality forms can influence cognitive and motor tasks performed by participants. In particular, participants were stimulated with requests made through a physical contact or vocally and conveying rude or gentle vitality forms, and then they were asked to estimate the end of a passing action observed in a monitor (action estimation task) or to perform an action in front of it (action execution task) with the intention to pass an object to the other person presented in the video. Results of the action estimation task indicated that the perception of a gentle request increased the duration of a rude action subsequently observed, while the perception of a rude request decreased the duration of the same action performed gently. Additionally, during the action execution task, accordingly with the perceived vitality form, participants modulated their motor response.

## Introduction

Social interactions are characterized by the capacity to communicate our intentions and affective states, and to evaluate those of others. This behavioral exchange is based on actions and speech dynamics of the interactants which have been defined by Daniel Stern with the term “vitality forms”^1,2^.

Vitality forms represent the way in which actions are performed and words are pronounced (the ‘’how’’) reflecting the agent’s affective state. Indeed, the same action, such as a handshake can be performed kindly or vigorously having a different impact on the receiver. Vitality forms play a fundamental role in social interactions, both from the agent and from the observer point of view. Indeed, these forms of communication provide the observer with essential indications to interact and better understand others. According to Stern (1985, 2010), actions, gestures, words and more generally, human behavior, are always characterized by vitality forms. In the absence of vitality forms, all actions would be similar and devoid of any affective component.

In recent years, fMRI studies have investigated the neural bases of these forms of communication, showing that the observation, imagination, and execution of actions conveying vitality forms induce the activity of the dorso-central insula^3–6^. Moreover, the same authors have demonstrated that listening to action verbs pronounced gently or rudely and imaging to pronounce the same verbal material with the same vitality forms activated the parieto-frontal circuit and the dorso-central insula^6^. While the activation of the parieto-frontal circuit indicated that individuals internally represented the listening actions communicated by verbs, the activation of insula showed that they also revived the forms of those actions. The activation of the insula during the perception and expression of vitality forms strongly suggests the existence of the mirror mechanism for vitality forms in the dorso-central insula. This mechanism could allow individuals from one side to understand vitality forms communicated by others remapping them on their internal motor schema, from the other to prepare an appropriate motor response^4,6^.

It is very plausible that, when interacting with others through actions execution and speech production, the gentle or rude vitality forms expressed by the agent may influence the affective state and the motor behavior of the receiver. In order to test this hypothesis, Di Cesare and colleagues (2017) carried out a kinematic study^7^. In this study, participants were presented with stimuli showing two requests (take it, give me) expressed gently or rudely and presented in visual, auditory, or mixed modality (visual and auditory). In accordance with the type of request (take it or give me), participants were required to take or give a bottle placed in front of them. Results showed that the gentle or rude vitality forms of the request influenced the individuals’ action execution. In particular, when participants received a rude request they interacted with the object using a higher velocity and a larger trajectory, while when they received a gentle request produced a soft interaction with the object, corresponding to a lower velocity and a smaller trajectory. These data clearly indicate that, during interpersonal interactions, vitality forms expressed by an agent affect the motor behavior of the receiver.

Human interactions are based on the capacity to perceive and perform vitality forms. An interesting issue is to understand whether, besides the action execution, vitality forms express by the agent may also affect the perception of the receiver. Indeed, since perception and execution of vitality forms based on the same neural circuit, in the present study we hypothesized that the vitality form of a request (give me) expressed physically and vocally may influence the internal representation of a subsequent action (passing an object), modifying some features such as its time duration. This hypothesis is in line with data provided by another psychophysical study recently carried out by our group^8^. For this purpose, we carried out a psychophysics experiment consisting of two different tasks. In the first task (Action Estimation Task), participants were presented with video clips showing the initial part of a passing action performed with different vitality forms (rude or gentle) and were asked to continue the action mentally (cognitive task) and estimate the time of its completion by pressing a button. Before the videos’ presentation, participants received two different types of stimulation: a request mediated by a physical contact, during which the robotic manipulandum reproduced a rude or gentle movement on their right arm, or a vocal request (“give me”) pronounced with a rude or gentle voice by two actors (a male and a female). In the second task (Action Execution Task), participants received a request mediated by a physical contact or vocally conveying rude or gentle vitality form and then were required to move actively the handle of the manipulandum with the aim to pass the object to another person showed on the monitor. We hypothesized an effect of gentle and rude vitality forms conveyed through vocal and physical requests (independent variables) on the estimation of action duration (dependent variable of the action estimation task) and on the kinematic parameters (i.e. velocity peak and distance covered) of the passing action performed by participants (dependent variables of the action execution task).

## Materials and methods

### Participants

The experiment was carried out on 18 healthy right-handed volunteers (twelve females and six males; mean age = 24.1 years; SD = 2.7 years). All participants were native Italian speakers and had normal or corrected-to-normal vision and normal hearing. None reported neurological or cognitive disorders. The study received approval by the ethical committee of Liguria Region (n.222REG2015) and was carried out according to the principles expressed in the Declaration of Helsinki. The participants provided written informed consent.

### Tasks and experimental paradigm

**Figure 1:**
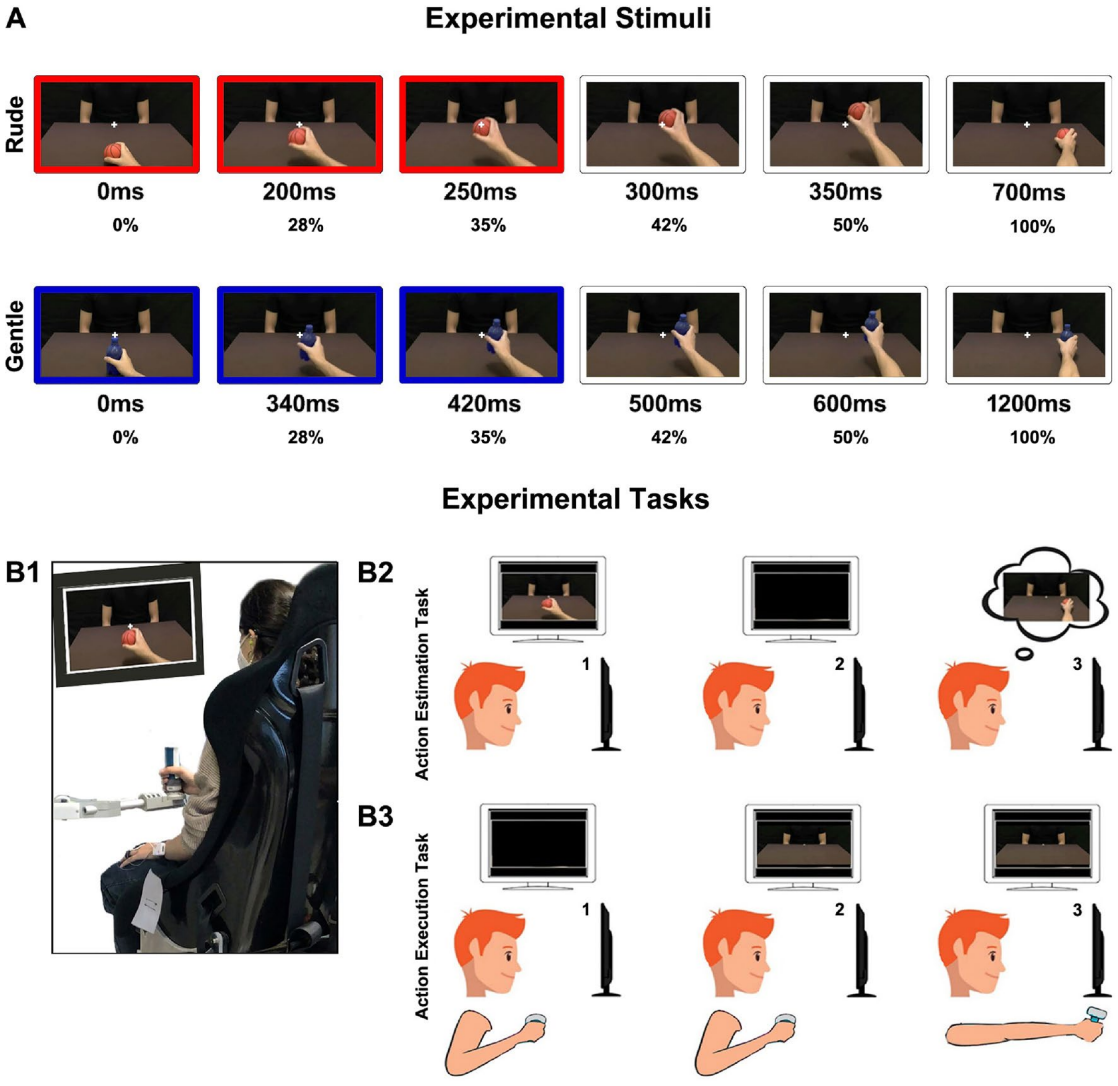
Visual stimuli (**A**): the total duration was respectively 700 ms for rude actions and 1200 ms for gentle actions. The stimuli consisted in presenting the 35% of the entire duration: 250 ms for rude actions (red color) and 420 ms for gentle actions (blue color). Example of a participant during the experiment (**B1**). *Action Estimation Task* (**B2**): (1) participants observed the initial part of a passing action, (2) the action was obscured, (3) they continued the action mentally estimating the time of its conclusion. *Action Execution Task* (**B3**): (1) starting position, (2) a static image of an actor appeared, (3) participants performed the passage moving the handle.

Participants sat in a comfortable chair in front of a monitor, holding the handle of the manipulandum “Braccio di ferro”^9^ with their right hand and wearing a pair of headphones (Fig. 1B1). The monitor was set to a spatial resolution of 1920 × 1080 pixels. The participants were required to perform two main tasks: an *Action Estimation Task* and an *Action Execution Task*. In the *Action Estimation Task* participants observed videos in which the right hand of an actor passed an object (a ball, a bottle, a cup or a packet of crackers) to another person represented on the other side of a table (Fig. 1A), with either a gentle or a rude vitality form.

**Figure 2:**
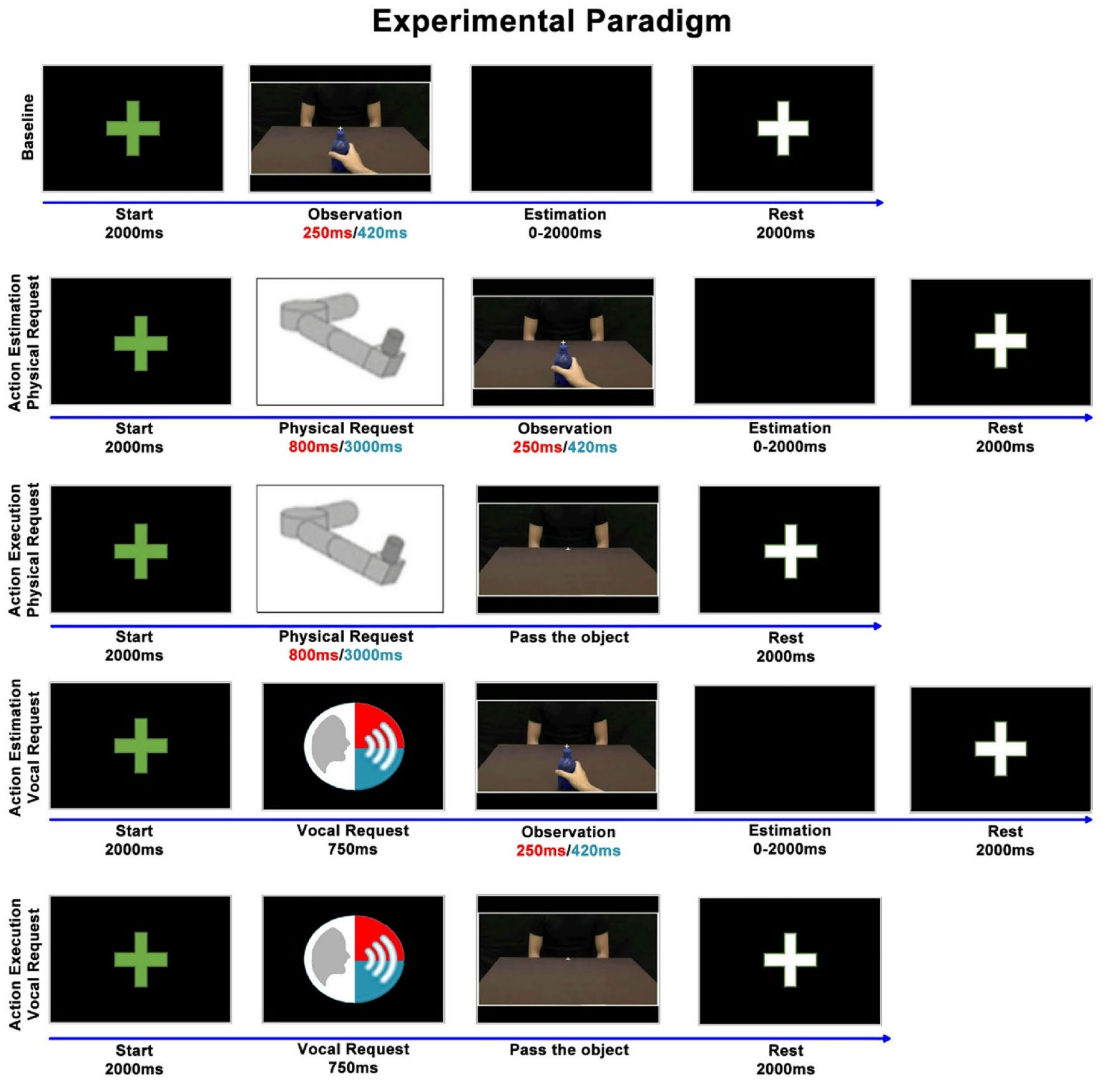
Experimental paradigm. For each run, the green fixation cross (Start, 2000 ms) indicated the beginning of a new trial. In the Baseline run and in the two Estimation runs (2nd and 4th rows) participants observed the initial part of the action (Observation), then the monitor turned black (Estimation, 0–2000 ms), and when they pressed the button on the handle to estimate the action conclusion a white fixation cross appeared (Rest, 2000 ms). In the two Action runs (3rd and 5th rows), when a static image of an actor/actress appeared (Pass the object), participants moved actively the handle in front of them. At the end of the passage, the manipulandum moved passively the arm at the starting position and the white fixation cross appeared (Rest, 2000 ms). The panel with the manipulandum icon (Physical Request) indicated that a physical request preceded the subsequent task. The panel with the audio icon indicated that a vocal request preceded the subsequent task. Red color corresponds to rude vitality forms while blue color corresponds to gentle vitality forms.

This egocentric perspective allowed participants to get involved in the action. More specifically, video stimuli consisted of showing only an initial part (35%) of the entire passing action, corresponding to 250 ms for rude actions (Fig. 1A, red color) and to 420 ms for gentle actions (Fig. 1A, blue color). After these durations, actions were obscured and participants were asked to continue them mentally, indicating the time of their conclusion by pressing a button located on the handle of the manipulandum (Fig. 1B2). In the *Action Execution Task* participants were instead presented with a static image of the same actors and were required to move actively the handle in front of them with the intention to pass an object. (Fig. 1B3). The experiment was composed of five runs (Fig. 2). In the Baseline run participants simply estimated the duration of actions. In the Estimation Physical Request run a physical request preceded the action estimation task: first, the manipulandum moved the participants’ arm gently or rudely and subsequently, they observed the initial part of the action and estimated its end. In the Action Physical Request run after receiving the physical request executed gently or rudely by the manipulandum, participants performed the action execution task, moving actively the handle towards the actor/actress. In the remaining runs requests were vocal. In particular, in the Estimation Vocal Request run participants listened to a voice pronouncing “give me” in a gentle or rude way and then they executed the action estimation task. Finally, in the Action Vocal Request run after receiving the vocal request expressed gently or rudely, participants performed the action execution task. With the purpose of avoiding a bias due to the runs order effect, the presentation order of experimental runs was balanced across participants. Nine participants started with the runs presenting a vocal request followed by the runs presenting a physical request. Nine participants started with the runs presenting a physical request followed by the runs presenting a vocal request. The stimuli presented randomly in the Baseline run were 24: 12 rude actions and 12 gentle actions, presented at the beginning of the experiment. In each of the two “Estimation” runs a random presentation of congruent and incongruent conditions was created to evaluate the influence of vitality forms characterizing the request on the action estimation task. This means that the requests (physical or vocal) and the following action stimuli could share the same vitality form (*rude* request—*rude* action or *gentle* request—*gentle* action) or could have opposite vitality form (*rude* request—*gentle* action or *gentle* request—*rude* action).

In particular, each of these runs presented 48 stimuli consisting of: 12 rude requests followed by rude actions (congruent condition), 12 rude requests followed by gentle actions (incongruent condition), 12 gentle requests followed by gentle actions (congruent condition), 12 gentle requests followed by rude actions (incongruent condition).

Finally, each of the two “Action” runs presented in a random order 24 stimuli: 12 rude requests and 12 gentle requests. Thus, we manipulated gentle and rude vitality forms (independent variables) to test their effect on the action estimated duration (dependent variable of the action estimation task) and the kinematic parameters of passing actions performed by participants (dependent variables of the action execution task).

For each run, a period of rest of two seconds was inserted between trials and it was marked by a white fixation cross on a black background to keep attention on the screen. Before the beginning of a new trial the white cross turned green. PsychoPy v3.0 software was used to present video stimuli and to record participants’ answers during the action estimation task. Physical requests were instead implemented and controlled through the software environment RT-Lab, integrated with MATLAB/Simulink. RT-Lab included a 100 Hz loop for data storage, which permitted to collect the hand trajectories during the action execution task. The kinematic data recorded were analyzed using MATLAB (R2020b). Particularly, action velocity was estimated by using a third order Savitzky–Golay smoothing filter. Action velocity curves were obtained considering the time interval between the end of each request (starting position, Fig. 1B3.1) and the moment in which participants completed their passing action (ending position, Fig. 1B3.3).

### Physical and vocal stimuli

**Figure 3:**
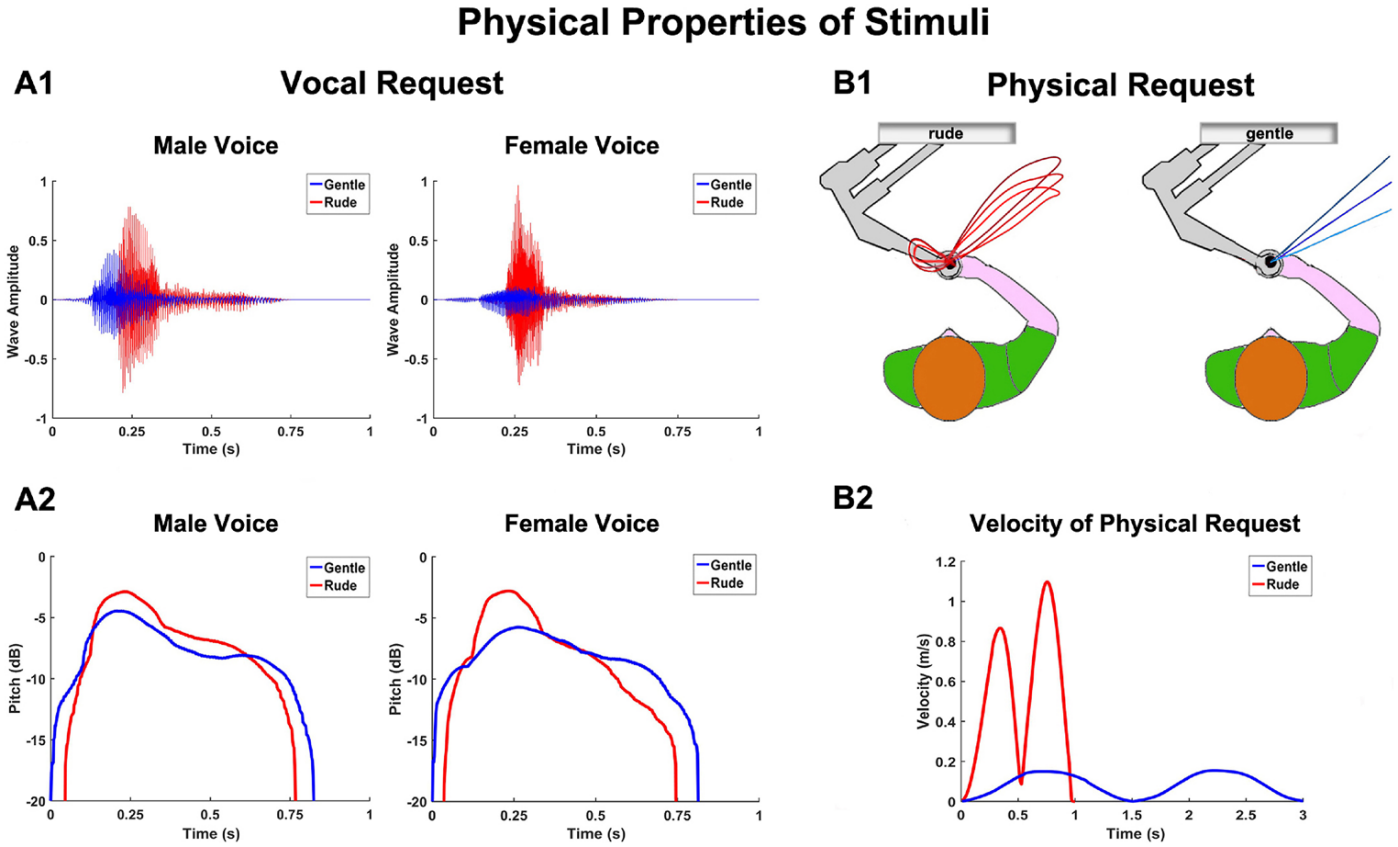
Physical properties of stimuli. Wave amplitude (**A1**) and pitch (**A2**) of rude (red) and gentle (blue) vocal requests for the male and the female voice. Spatial trajectories of the motions performed by the manipulandum to provide rude (red lines) or gentle (blue lines) physical requests (**B1**). Velocity module of rude (red line) and gentle (blue line) physical requests (**B2**).

As described in the previous section, participants could be physically or vocally stimulated before performing action duration estimation or action execution tasks. The physical parameters (velocity and trajectory) used to implement the physical request were derived from a previous kinematic recording in which an actor was asked to move the handle in a rude or gentle way. This procedure allowed us to generate a robotic movement that faithfully reproduced human vitality forms (rude and gentle). The stimuli performed through the manipulandum consisted of displacements on the horizontal plane starting from the coordinate (0 m, − 0.1 m) of the workspace. For each physical request, the arm of participants was moved in the right direction and returned to the starting position. This robotic movement was completely different in terms of direction from those observed/executed by participants, avoiding a possible motor imitation effect between physical requests (frontal direction) and participants’ movements (rightward). Rude and gentle requests were presented in a random order and differed for trajectory and velocity. Additionally, in order to exclude a phenomenon of adaptation, for each vitality (rude and gentle) the manipulandum performed three movements with the same velocity (Fig. 3B2), but with a small angular shift among them (− 10°, 0°, + 10°; Fig. 3B1).

The rude request lasted 800 ms with a maximum displacement of 22 cm in the x-direction (right side). In contrast, the gentle request lasted 3000 ms with a maximum displacement of 10 cm in the same x-direction. On the other hand, during the vocal request participants listened to a male or a female voice pronouncing the Italian verb “dammi” (“give me”) in either a rude or a gentle way. Each vocal request was recorded using a condenser microphone (RODE NT1) placed 30 cm in front of the actors and digitized with a phantom powered A/D converter module (M-AUDIO M-TRACK). After recording, the audio files were processed with COOL EDIT PRO software. Rude and gentle vocal requests differed for parameters such as the wave amplitude and the pitch (Fig. 3A1, A2).

## Results

### Action estimation task

**Figure 4:**
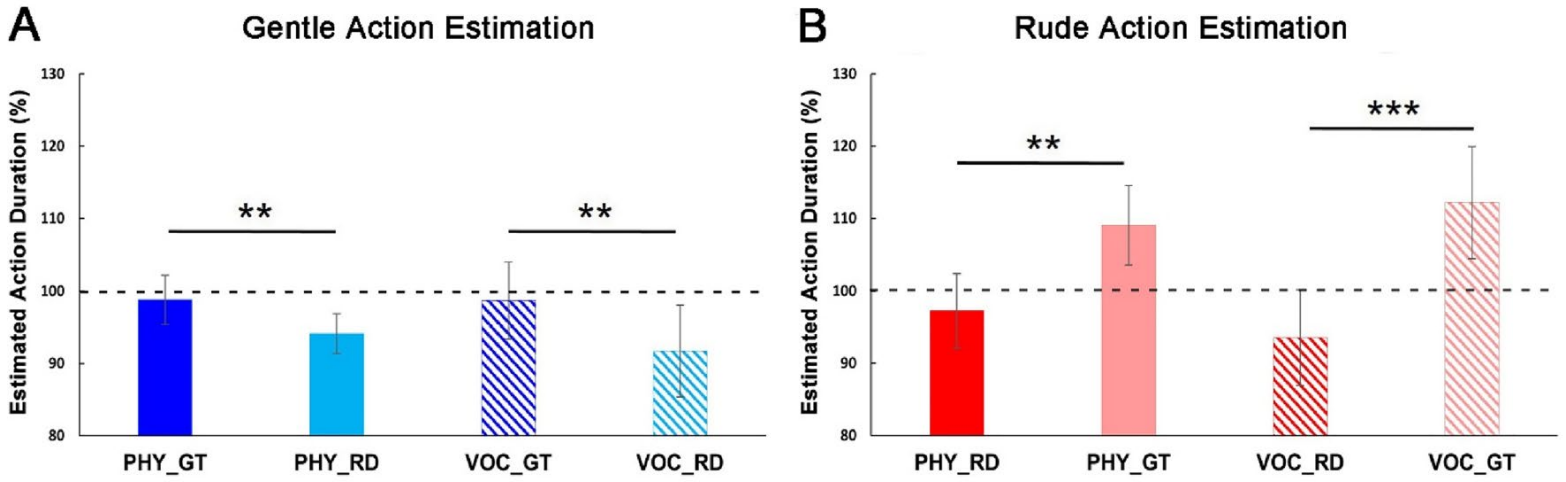
Results of gentle (**A**) and rude (**B**) action estimation. Estimated action durations normalized to the baseline condition are reported on the y-coordinate. The dotted line in correspondence of 100% refers to the baseline. *PHY* physical request, *VOC* vocal request, *GT* gentle vitality form, *RD* rude vitality form. Vertical bars represent the standard errors (SE). Horizontal bars indicate statistical significance (**p < 0.01, ***p < 0.001). Effect of rude requests on gentle action estimation.

Considering the participants’ responses (estimated action duration) we measured the presence of possible differences between rude and gentle requests for both physical and vocal modalities. Specifically, the participants’ responses obtained after physical and vocal requests were normalized to the baseline condition (Estimated action duration (%) = estimated action duration after request *100/estimated action duration during baseline condition), obtaining percentage values as shown in Fig. 4. Then, four paired sample t-tests (two for gentle action estimation and two for rude action estimation) were carried out to assess possible differences between congruent and incongruent conditions, after physical (PHY) or vocal (VOC) requests. The significance level was fixed at *p* = 0.05. Before performing statistical analysis, the sphericity of data was verified (Mauchly’s test, *p* > 0.05). All variables were normally distributed (Kolmogorov–Smirnov Test, *p* > 0.05). Results showed a significant difference between congruent and incongruent conditions, for both gentle and rude vitality forms, regardless of the type of request (*p* < 0.05, Fig. 4A, B; for details see also supplementary material).

### Action execution task

**Figure 5:**
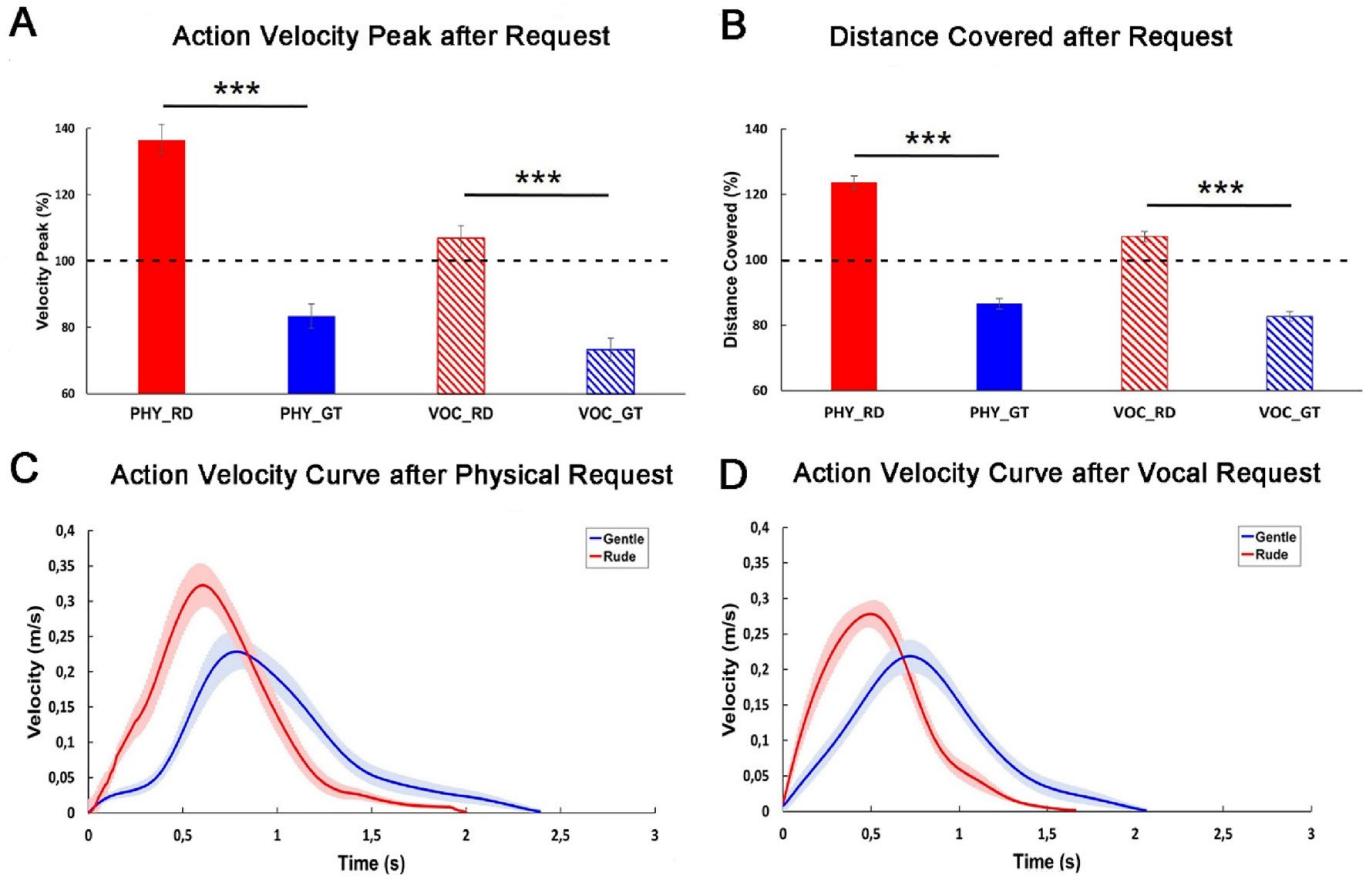
Action velocity peak (**A**) and distance covered (**B**) after request normalized to the baseline condition. Red color refers to rude requests (RD) and blue color refers to gentle requests (GT), both expressed physically (PHY) or vocally (VOC). Graphs in the middle show the action velocity curve characterizing the passage performed by participants after a physical (C) or a vocal (D) request. Error shading indicate standard error of the mean.

The action parameters characterizing the passage performed by participants after physical and vocal requests were normalized to the baseline condition as described above, obtaining percentage values as shown in Fig. 5A and B. Then, four paired sample t-tests (two for action velocity peak and two for distance covered after requests) were carried out to assess possible differences between actions performed by participants after physical (PHY) or vocal (VOC) requests. Results showed a significant difference between actions performed after rude and gentle requests, regardless the type of request (*p* < 0.001 as shown in Fig. 5A, B; for details see also supplementary material). This difference is also highlighted in Fig. 5C and D, which shows the mean action velocity curves of participants in response to gentle and rude requests (physical and vocal).

## Discussion

Vitality forms represent a fundamental aspect of social communication allowing people to express their mood/attitude and to understand immediately those of others. For example, according to a positive or negative mood of the agent, an action can be performed as gentle or rude towards another individual. In spite of their pervasiveness in human interactions, the influence of vitality forms on action perception has never been addressed. In this view, the aim of the present study was twofold: 1) to investigate whether and how vitality forms expressed physically or vocally by an agent may affect the participants’ responses during a cognitive task (action estimation task); 2) to assess how the same requests may modulate the kinematic parameters of a subsequent action (passing an object; action execution task). Results indicated that, during the action estimation task, a gentle request, independently of its modality (through physical contact or vocally) increased the duration of the subsequent action observed by participants. In contrast, a rude request affected the perception of an action subsequently presented, decreasing its perceived duration. More specifically, when participants observed the initial part of a gentle action but were previously stimulated with a rude request (incongruent condition), they anticipated the end of this action compared to the same action presented during the congruent condition (gentle request, gentle action). On the other hand, when they observed the initial part of a rude action but were previously stimulated with a gentle request (incongruent condition**)**, they perceived this action as lasting longer compared to the same action presented in the congruent condition (rude request, rude action). Moreover, results of the action execution task indicated that, for both physical and vocal requests, the perception of vitality forms modulated the kinematic parameters (i.e., velocity and distance covered) of the subsequent action performed by participants. In particular, after a rude request, their action (passing the object) had a higher velocity peak and covered a bigger distance. In contrast, after a gentle request, the same action was performed with a lower velocity peak and covered a smaller distance.

Taken together, these findings highlight the important role of vitality forms during social interactions, showing how a simple request expressed gently or rudely influence the perception and the motor behavior of the receiver. Interestingly, this modulation effect played by vitality forms on the receiver also occurred when participants listened to the vocal request, suggesting that this influence cannot be merely ascribed to a motor imitation mechanism. These findings are in line with data provided by two psychophysical studies recently carried out by our group^8^ where participants listened to gentle/rude vocal requests and then observed the initial part of a passing action consisting in different durations (200 ms, 250 ms, 300 ms, 350 ms for rude actions; 340 ms, 420 ms, 500 ms, 600 ms for gentle actions). As in the present study, once the action was obscured, participants were required to continue it mentally, indicating its end (action estimation task of the present study)^8^. Results showed that listening to rude/gentle vocal requests influenced the perception of actions subsequently observed. In addition, the same authors quantified the duration of this effect adding five time delays (0 ms, 200 ms, 400 ms, 800 ms, 1200 ms, 1600 ms) between the vocal request and the video’s presentation, finding that the effect lasted 800 ms and then started to decay.

All together, these findings suggest that vitality forms expressed by an agent automatically influence the perception of a subsequent action observed by the receiver. An interesting question is to understand how it is possible. When an individual observes an action performed by other individuals, he/she is able to understand their action-goal as well as their intentions. This ability depends on the existence of a brain network named action-observation network (AON) selectively activated during the observation of arm/hand actions. This AON network consists of three bilateral cortical areas that are reciprocally connected: the ventral premotor cortex, inferior parietal lobule and superior temporal sulcus^10,11^. In the last years, several fMRI studies carried out by our group showed that the observation of hand action performed with different vitality forms (gentle/rude) besides the activation of AON network produced the activation of the dorso-central insula^3–7,12–16^. Notably, this cortical area is involved during both the observation and the execution of different vitality forms. Tract-tracing investigations carried out in monkeys and humans showed that the dorso-central insula is connected with the arm-and-hand control circuit (AON)^12^. In this view, it is plausible that this area may transforms the vitality form information into a motor domain allowing individuals, from one side to understand vitality forms expressed by the agent, from the other, to prepare an adequate response to a subsequent action.

One may hypothesize that the affective contagion of the vocal request on the action perception may be ascribed to a potential arousal effect. Specifically, it is easy to assert that listening to a rude voice conveying a rude request may induce the receiver to assume an alert state, making his response faster. However, this hypothesis is not supported by results obtained during the estimation of gentle actions. Indeed, although participants were required to estimate the duration of rude actions, the gentle request induced them to estimate the actions as lasting longer. This view is also corroborated by recent findings showing that the effect of gentle vocal request on rude action estimation was significantly greater than the effect of rude vocal request on gentle action estimation indicating that the effect of vitality forms on action estimation was not merely due to an arousal effect^8^.

Notably, during social interactions, the agent can communicate his attitude to the receiver in different ways such as facial expressions, body postures and of course the voice tone. In contrast, in the present study participants perceived only a vocal request pronounced gently and rudely. This limitation could have minimized the effect of the vitality forms on the participants. Indeed, in a realistic scenario, it is plausible that the vitality forms effect may have a stronger impact on the receiver than that found in our study. Another important limitation concerns the fact that the experiment was carried out on European individuals. Indeed, it is plausible that people from different cultures may express positive and negative attitudes towards others in different ways. For example, Caucasian, Asian, and African people are characterized by a different tone of voice that could have a different impact on the receiver.

A strong point of this research is that it highlights that actions and pronounced words are characterized by two distinct components: the content (goal) and the form (how the goal is achieved). While the most part of previous studies, for the best of our knowledge, aimed to investigate the action goal (what), our study represents the first attempt to assess the ability in encoding the action form (how). Specifically, our results indicate that observing a very small part of an action, the observer is able to capture immediately the vitality form of that action. It is plausible that, during the task, participants processed the action kinematic information and remapped them on their own motor schema. This remapping process have probably permitted to represent internally the vitality forms of the observed action, preparing an adequate response^17–20^.

Findings provided by the present study on the influence of vitality forms on others, extend the knowledge on the role of the affective states on the affective contagion of others. Previous studies showed how the perception of facial mimicry^21–24^, body postures^25^, and vocalizations^26,27^, produced in the receiver an affective convergence supporting the existence of an automatic mechanism selective for the affective contagion. In a recent study carried out by Pinilla and colleagues^28^ indicated that when participants were induced to a positive mood/affective state, they judged both happy and angry faces closer to a positive affective state. In contrast, when participants were induced to a negative mood/affective state, they judged both happy and angry faces closer to a negative affective state.

Interestingly vitality forms can be effective also when expressed by non-human agents. Drawing inspiration from human voice and movement, a recent study^29^ modified a passing action and a voice generated by the humanoid robot iCub, with the aim of transmitting vitality forms. Results showed that the kinematic parameters of the robot's movement and the properties of its voice are adequate to express different attitudes, which are consistently perceived as rude or gentle by human partners. Moreover, the same authors showed that the observation of robotic actions conveying vitality forms (rude and gentle) produced an increase of the BOLD signal in the dorso-central insula, the same sector activated during the perception of vitality forms expressed by humans^30^.

In conclusion, our data shed new light on the fundamental role of vitality forms in social interactions. We clearly demonstrated how physical and vocal requests conveying different vitality forms modulate the response of the receiver. In particular, when an individual asks us something, his/her positive and negative attitudes, communicated by vitality forms, modulate both our perception and motor behavior. The role of vitality forms in influencing others from an affective state point of view highlights their relevance in social communication. Results and methodology from this study may have implications for social and communicative disorders and other research fields such as robotics.

## Supplementary Information

Supplementary Information.
