# Supplementary material for: The influence of vitality forms on action perception and motor response
Source: Sci Rep. 2021 Nov 19;11:22576. doi: 10.1038/s41598-021-01924-w (PMC8605011; doi:10.1038/s41598-021-01924-w)
Supplement: Supplementary file 1 — Supplementary Information. [file 41598_2021_1924_MOESM1_ESM.docx]

**REVISIONS**

**Action Estimation Task**


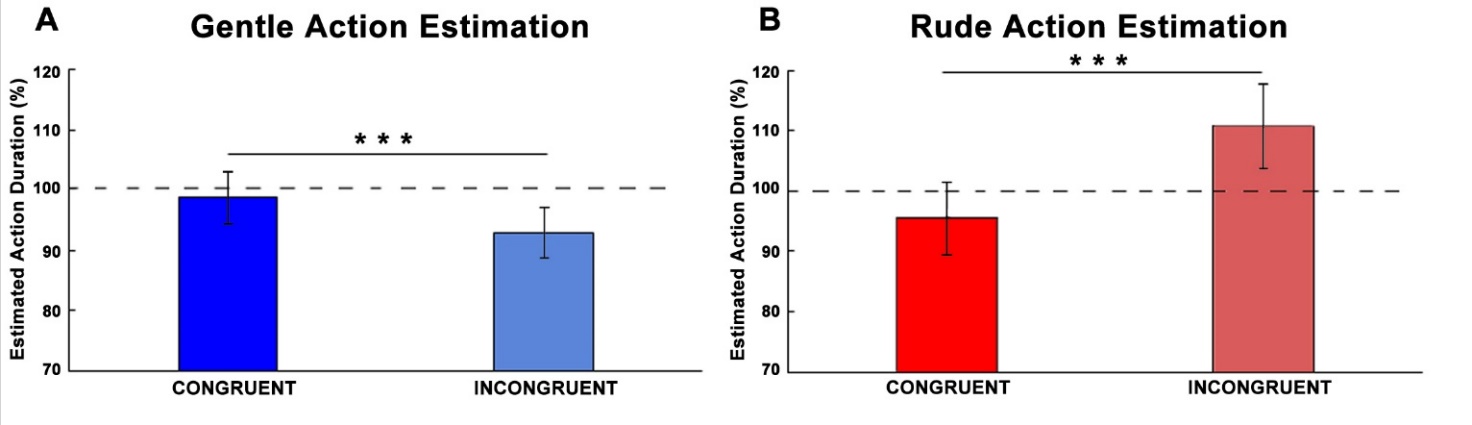
The participants’ responses obtained after physical and vocal requests were normalized to the baseline condition (action estimation time (%) = action estimation time after request *100 / action estimation time during baseline condition), obtaining percentage values as shown in Figure S1. The significance level was fixed at p = 0.05. Before performing statistical analysis, sphericity of data was verified (Mauchly’s test, p > 0.05). All variables were normally distributed (Kolmogorov-Smirnov Test, p > 0.05). In order to assess a possible effect of the type of request (physical/vocal) and of the experimental condition (congruent/incongruent) on the action estimation time, we carried out two Repeated Measured GLM, one for gentle and one for rude vitality forms. Results revealed a significant difference between congruent and incongruent conditions (gentle action estimation: congruent = 99%, incongruent = 93%; F(1,17) = 14.5, p = 0.001, see Figure S1A; rude action estimation: congruent = 95%, incongruent = 110%; F(1,17) = 19.27, p < 0.001, see Figure S1B). No significant difference between physical and vocal requests and no significant interaction (Request * Congruence) were found.

**Figure S1:** Indipendently from the modality through which the request was expressed, results of GLM revealed a significant difference between cogruent and incongruent conditions, for both gentle action estimation (A) and rude action estimation (B). The dotted line in correspondence of 100% refers to the baseline. Vertical bars represent the standard errors (SE). Horizontal bars indicate statistical significance (***p < 0.001).

**Action Execution Task**

The action’s parameters characterizing the passage performed by participants after physical and vocal requests were normalized to the baseline condition as described above, obtaining percentage values as shown in Figure 5. In order to assess a possible effect of requests (physical/vocal) and vitality forms conveyed by the same requests (rude/gentle) on the action execution, we carried out two Repeated Measured GLM, one for action velocity peak and one for distance covered after requests. Results revealed a significant difference between physical and vocal requests (action velocity peak: F(1,17) = 17.84, p = 0.001, distance covered: F(1,17) = 34.04, p < 0.001) and a significant difference between rude and gentle vitality forms conveyed by requests (action velocity peak: F(1,17) = 60.39, p < 0.001; distance covered: F(1,17) = 138.7, p < 0.001). Finally, a significant interaction between the two factors was found (action velocity peak: F(1,17) = 9.38, p = 0.007; distance covered: F(1,17) = 33.78, p < 0.001). Post hoc analysis revealed a significant difference between all the conditions (p < 0.001, Bonferroni corrected) except for the comparison between gentle physical request (PHY_GT) and gentle vocal request (VOC_GT), (action velocity peak: p = 0.2; distance covered: p = 0.138; see
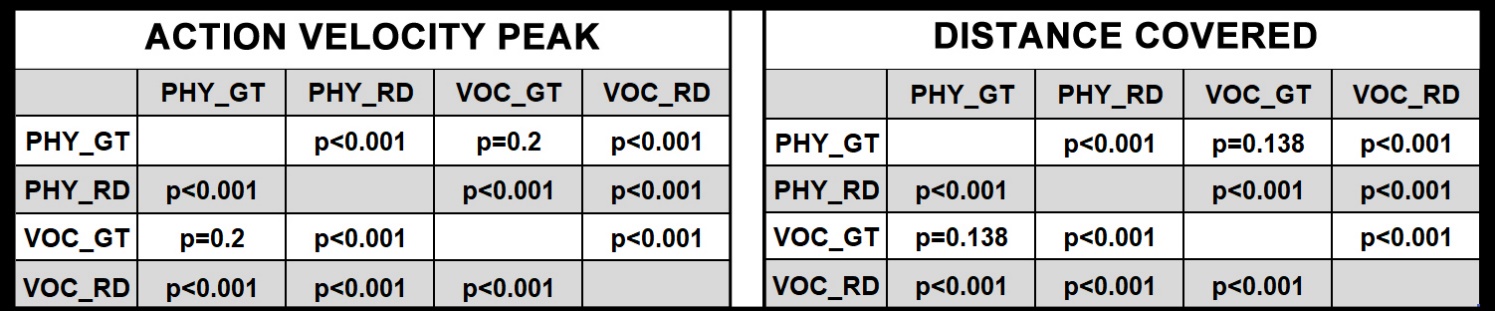
Table1).

**Table1:** Post hoc analysis relative to the action execution task revealed a significant difference between all the conditions (***p < 0.001) except for the comparison between PHY_GT (gentle physical request) and VOC_GT (gentle vocal request), for both action velocity peak (p = 0.2) and distance covered (p = 0.138).
